# The Prognostic Significance of Eukaryotic Translation Initiation Factors (eIFs) in Endometrial Cancer

**DOI:** 10.3390/ijms20246169

**Published:** 2019-12-06

**Authors:** Maria Anna Smolle, Piotr Czapiewski, Sylwia Lapińska-Szumczyk, Hanna Majewska, Anna Supernat, Anna Zaczek, Wojciech Biernat, Nicole Golob-Schwarzl, Johannes Haybaeck

**Affiliations:** 1 Department of Orthopaedics and Trauma, Medical University of Graz, Auenbruggerplatz 5, 8036 Graz, Austria; maria.smolle@medunigraz.at; 2 Area 2 Cancer, Center for Biomarker Research in Medicine, Stiftingtalstraße 5, 8010 Graz, Austria; Nicole.golob@medunigraz.at; 3 Department of Pathomorphology, Medical University of Gdansk, Mariana Smoluchowskiego 17, 80-214 Gdańsk, Poland; czapiewskipiotr@gumed.edu.pl (P.C.); hania.majewska@gumed.edu.pl (H.M.); biernat@gumed.edu.pl (W.B.); 4 Department of Pathology, Medical Faculty, Otto-von-Guericke University Magdeburg, Leipziger Straße 44, 39120 Magdeburg, Germany; 5 Department of Gynaecology, Gynaecological Oncology and Gynaecological Endocrinology, Medical University of Gdańsk, M. Skłodowskiej-Curie 3a Street, 80-210 Gdańsk, Poland; slapin@wp.pl; 6 Department of Medical Biotechnology, Intercollegiate Faculty of Biotechnology, University of Gdańnsk and Medical University of Gdańsk, Bażyńskiego 1a, 80-952 Gdańsk, Poland; anna.supernat@biotech.ug.edu.pl (A.S.); azaczek@gumed.edu.pl (A.Z.); 7 Institute of Pathology, Medical University of Graz, Auenbruggerplatz 25, 8036 Graz, Austria; 8 Department of Pathology, Neuropathology and Molecular Pathology, Medical University of Innsbruck, Müllerstraße 44, 6020 Innsbruck, Austria

**Keywords:** eukaryotic translation initiation factors, endometrial cancer, prognostic marker

## Abstract

Whilst the role of eukaryotic translation initiation factors (eIFs) has already been investigated in several human cancers, their role in endometrial cancer (EC) is relatively unknown. In the present retrospective study, 279 patients with EC (1180 samples) were included (mean age: 63.0 years, mean follow-up: 6.1 years). Samples were analysed for expression of 7 eIFs subunits (eIF2α, eIF3c, eIF3h, eIF4e, eIF4g, eIF5, eIF6) through immunohistochemistry and western blotting. Fifteen samples of healthy endometrium served as controls. Density and intensity were assessed and mean combined scores (CS) calculated for each patient. Upon immunohistochemistry, median eIF5 CS were significantly higher in EC as compared with non-neoplastic tissue (NNT, *p* < 0.001), whilst median eIF6 CS were significantly lower in EC (*p* < 0.001). Moreover, eIF5 (*p* = 0.002), eIF6 (*p* = 0.032) and eIF4g CS (*p* = 0.014) were significantly different when comparing NNT with EC grading types. Median eIF4g CS was higher in type II EC (*p* = 0.034). Upon western blot analysis, eIF4g (*p* < 0.001), peIF2α (*p* < 0.001) and eIF3h (*p* < 0.05) were significantly overexpressed in EC, while expression of eIF3c was significantly reduced in EC as compared with NNT (*p* < 0.001). The remaining eIFs were non-significant. Besides tumour stage (*p* < 0.001) and patient’s age (*p* < 0.001), high eIF4g CS-levels were independently associated with poor prognosis (HR: 1.604, 95%CI: 1.037–2.483, *p* = 0.034). The other eIFs had no prognostic significance. Notably, the independent prognostic significance of eIF4g was lost when adding tumour type. Considering the difficulties in differentiating EC type I and II, eIF4g may serve as a novel prognostic marker indicating patient outcome.

## 1. Introduction

Endometrial cancer (EC) is the fifth most common cancer in females and the most frequently diagnosed gynaecological malignancy in developed countries [1]. The mean age of patients at the time of diagnosis is 63 years [2]. Primary risk factors include diabetes, alcohol abuse, a history of infertility and elevated oestrogen levels due to obesity, polycystic ovary syndrome and oestrogen use [3]. Early-stage EC is limited to the uterus and associated with a rather good prognosis. However, the increasing incidence of non-endometrioid subtypes, high-grade tumours and advanced-stage ECs at the time of diagnosis has led to a doubling of EC-related deaths over the last 30 years [4].

Patients commonly notice abnormal uterine bleeding, with hysteroscopy and biopsy subsequently confirming the suspected diagnosis [5,6]. However, a negative endometrial sampling may not rule out underlying malignancy and should prompt further investigation [6].

Historically, ECs were subdivided into two groups based on the pathogenesis and histological presentation [7]. Type I ECs arise in an oestrogen-rich environment on the basis of endometrial hyperplasia, express hormone receptors, present with a well-differentiated endometrioid histological pattern and are associated with good prognosis. On the other hand, type II ECs develop from atrophic endometrium, are not associated with oestrogen excess, have a non-endometrioid differentiation (i.e., serous, clear cell) and carry a worse prognosis compared to type I EC [7]. Over the last years, the characterization of EC based on molecular features has gained significance. In parallel, the role of type I and II EC has become less important, since molecular features frequently overlap between the two types [8]. Mutations in the *PIK3CA* genes are found in about half of serous and endometrioid carcinomas [9]. In nearly 90% of ECs with serous differentiation, inactivating *TP53* mutations are present, and about 80% of endometrioid carcinomas harbour inactivating *PTEN* mutations [9,10]. Consequently, mutations in the *TP53* gene are thought to drive the development of serous subtypes, whilst a *PTEN* loss is rather causative of endometrioid carcinoma [11]. Moreover, non-coding RNAs, a family of non-protein coding RNA sequences, may also be involved in the pathogenesis of EC, considering that many of them are aberrantly expressed in comparison to healthy endometrium [12,13].

The most important prognostic factor in surgical stage, followed by tumour size, grade, subtype and patients’ age [14]. Importantly, the addition of molecular features further aids outcome estimation [10].

In recent years, the role of eukaryotic translation initiation factors (eIFs) in carcinogenesis has been steadily uncovered [15,16,17]. They are involved in tumour development, progression and invasion. eIFs mainly regulate the classic—or canonical—process of translation initiation and can have oncogenic or tumour-suppressive functions [15]. Twelve core eIFs are involved in translation initiation, namely eIF1, eIF1a, eIF2, eIF2b, eIF3, eIF4h, eIF4a, eIF4e, eIF4g, eIF4b, eIF5 and eIF5b [15]. The latter three eIFs form the heterotrimeric eIF4F complex, which mediates the ribosomal recruitment to RNA as the rate-limiting process in translation initiation [18]. Moreover, eIFs are involved in cell cycle regulation and interact with important tumour-promoting pathways, including mTOR- and NF-kB-signalling [15,19,20].

The aim of this study was to investigate the role of seven eIFs subunits in endometrial cancer. We sought to correlate eIF expression levels with clinicopathological features and to evaluate their prognostic significance.

## 2. Results

### 2.1. Correlation between Clinicopathological Features and eIF Expression

Basic demographic and pathological features are listed in Table 1. The mean patients’ age was 63.0 years (standard deviation (SD): 10.6 years), with 117 patients (41.9%) younger than 60 years at the time of diagnosis.

**Figure 1:**
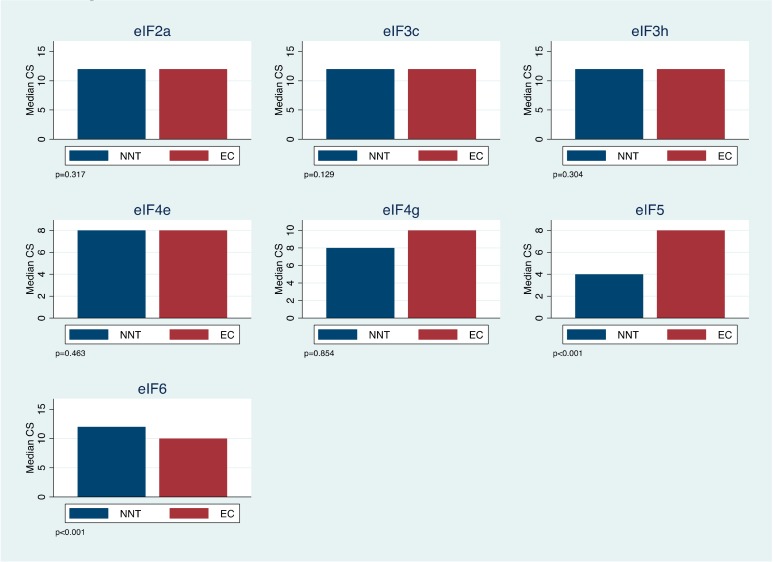
eIF combined score (CS) expression for non-neoplastic tissue (NNT) in comparison to endometrial cancer (EC). CS was stratified into low (≤11) and high (>11).

The seven eIFs showed a strong correlation among each other (Supplementary Table S1). Median eIF5 combined scores (CS) was significantly higher in EC as compared with NNT (8 (interquartile range (IQR): 5–10.7) vs. 4 (IQR: 4–4). U-test, *p* < 0.001). On the other hand, eIF6 CS was significantly lower in EC in comparison to NNT (10 (IQR: 8–12) vs. 12 (IQR: 12–12) U-test, *p* < 0.001). The CSs of the remaining eIF subunits did not significantly differ between NNT and EC (Table 2 and Table 3, Figure 1).

Moreover, whilst there was a significant difference for eIF5 (Kruskal-Wallis-test, *p* = 0.002), eIF6 (Kruskal-Wallis-test, *p* = 0.032) and eIF4g (Kruskal-Wallis-test, *p* = 0.014) CSs between NNT and G1, G2 and G3 EC, there was no significant difference in the CSs of the remaining eIFs (Table 2 and Table 3). Interestingly, eIF4g CS only showed significantly lower levels for EC type I in comparison to EC type II (10 (IQR: 6.7–12) vs. 11 (IQR: 9.75–12), *U*-test, *p* = 0.034). The remaining eIF subunit CSs were not significantly different depending on the EC type (Table 2 and Table 3).

Corresponding to the observation that eIF5 and eIF6 CSs showed significant differences between NNT and EC grading types, there was also a significant difference between NNT and EC staging groups (NNT vs. I vs. II vs. III vs. IV, Kruskal-Wallis-test, eIF5: *p* = 0.004, eIF6: *p* = 0.020, Table 2 and Table 3).

### 2.2. eIF Expression Is A Marker in Endometrial Cancer

We further investigated the expression patterns of peIF2α, eIF2α, eIF3c, eIF3h, eIF4e, eIF4g, eIF5 and eIF6 in endometrial cancer compared to non-neoplastic patient samples using immunoblot analysis.

**Figure 2:**
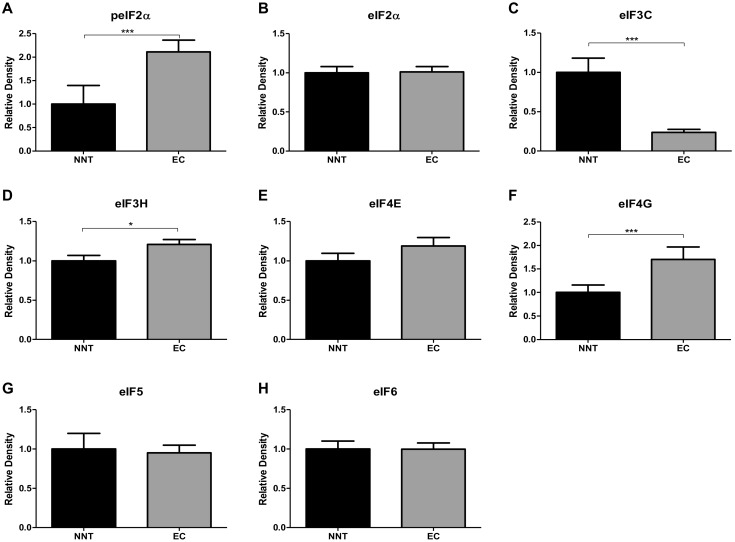
eIF protein expression in endometrial carcinoma was compared with non-neoplastic tissue (NNT) using immunoblot analyses. Alterations in the protein expression pattern of peIF2α (**A**), eIF2α (**B**), eIF3C (**C**), eIF3H (**D**), eIF4E (**E**), eIF4G (**F**), eIF5 (**G**) and eIF6 (**H**) were observed. Densitometric analyses of immunoblots using ImageJ software (NIH, MD, United States) were performed. Relative densities were normalized to Actin as the loading control. T- and Mann–Whitney *U*-tests were performed for statistical analysis, * *p* < 0.05 and *** *p* < 0.001.

The protein expression levels of peIF2α (Figure 2A and Supplementary Figure S1A,B), eIF3h (Figure 2D) and eIF4g (Figure 2F and Supplementary Figure S1A,B) were significantly increased in EC patients compared to NNT. The protein expression level of the eIF3c (Figure 2C and Supplementary Figure S1A,B) was significantly decreased in EC compared to NNT. Furthermore, eIF2α (Figure 2B and Supplementary Figure S1A,B), eIF4e (Figure 2E and Supplementary Figure S1A,B), eIF5 (Figure 2G and Supplementary Figure S1A,B) and eIF6 (Figure 2G and Supplementary Figure S1A,B) displayed no differences in the protein expression between EC and NNT.

### 2.3. Prognostic Significance of eIF Expression

**Figure 3:**
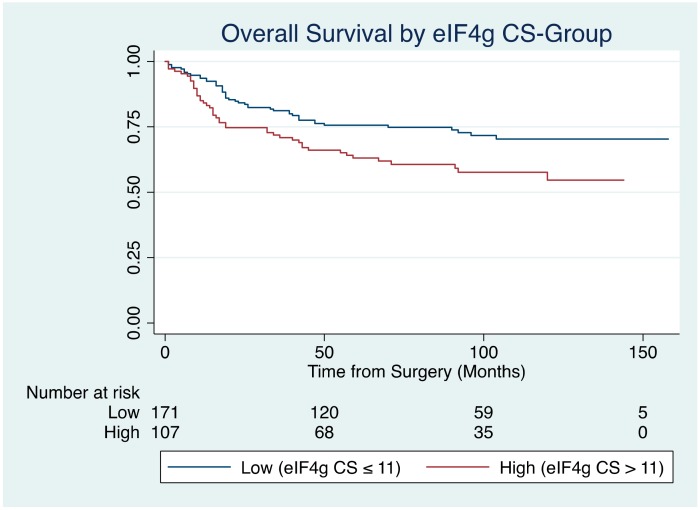
Kaplan Meier survival curve showing the influence of eIF4g combined score (CS) groups on overall survival in patients with endometrial cancer (*p* = 0.013).

The mean follow-up period after surgery was 6.1 years (SD ± 3.7 years). Five- and 10-year survival rates for all patients were 71.5% and 65.0%, respectively. As defined in the Methods section of the manuscript, CSs were subdivided into “high” (CS > 11) and “low” (CS ≤ 11) staining groups. Subsequently, in the univariate analysis for overall survival, a high eIF4g CS was associated with poor patient prognosis (HR (hazard ratio): 1.687, 95%CI (95% Confidence Interval): 1.116-2.550. *p* = 0.013, Figure 3).

In other words, 3- and 5-year OS was 81.7% and 76.2% for the eIF4g “low” staining group, in comparison to 71.4% and 63.8% for the “high” staining group, respectively. For the remaining eIF “high” and “low” CSs, no such association was found (Table 4).

Based on these observations, we sought to further investigate the independent prognostic significance of eIF4g in EC. Besides staging (*p* < 0.001) and patient’s age at the time of diagnosis (*p* < 0.001), high eIF4g CS levels were independently associated with poor prognosis (HR: 1.604, 95% CI: 1.037-2.483, *p* = 0.034, Table 5). Of note, there was no statistically significant difference in age between patients with low and high eIF4g CS levels (63.1 ± 10.4 vs. 62.6 ± 11.1, *t*-test, *p* = 0.702).

Notably, by adding EC type to the multivariate model, the independent prognostic significance of eIF4g was lost.

## 3. Discussion

In the present study, the prognostic significance of the eIFs 2α, 3c, 3h, 4e, 4g, 5 and 6 in endometrial cancer was investigated. Upon IHC (using combined scores, (CS)), eIF5 expression was significantly higher in EC in comparison to NNT, whilst eIF6 expression was expressed at significantly lower levels in EC as compared with NNT. Furthermore, expression levels of eIF5, eIF6 and eIF4g were significantly different between NNT and EC grading types. Western blot analysis showed no significant difference in eIF5 or eIF6 expression between EC and NNT, whilst eIF4g levels were significantly higher, as were eIF2α and eIF3h levels. Moreover, eIF4g levels were more frequently found in type II EC in comparison to type I EC and were independently associated with a worse patient survival, irrespective of tumour stage and the patients’ age at the time of diagnosis.

Limitations of the present study include its retrospective design, resulting in a heterogeneous cohort of patients at different tumour stages assigned to variant treatments. Furthermore, the amount of NNT samples was smaller than calculated by power-analysis. We approached these issues by including patient age and stage in the multivariate model to assess the prognostic significance of eIFs. Furthermore, IHC and western blot analysis revealed contradictory results for all but one eIF (eIF4g) regarding the expression between NNT and EC. Therefore, eIF4g was further investigated for its prognostic significance.

Whilst the importance of eIFs in different human cancers is well established [15], little is known about the role of eIFs in EC. So far, only one study has investigated the prognostic significance of eIF4e in EC [21]. According to Choi et al., eIF4e-positivity was associated with advanced tumour stage (III and IV) and reduced patient prognosis [21]. Other gynaecological malignancies, including cervical cancer [22,23,24,25], ovarian carcinoma [19,26,27,28,29] and breast cancer [30,31,32], have been investigated more thoroughly regarding the prognostic, predictive and therapeutic relevance of eIFs. In cervical cancer, overexpression of eIF5a2 as detected by immunohistochemistry correlates with poor patient prognosis [25]. Likewise, strong staining of eIF4e is associated with high-grade cervical cancer [24]. In the present study, though, eIF4e expression did not correlate with clinicopathological features or patient prognosis.

In benign pathologies of the endometrium, such as endometriosis, aberrant eIF expression may likewise be present. In endometriosis, for example, eIF3e-levels are reduced in comparison to healthy endometrium and do correlate with overexpression of E-Cadherin [33]. Therefore, eIF3a may play a role in the epithelial-to-mesenchymal transition [33]. In our study, no significant alteration in the expression of eIF3 subunits b (i.e., eIF3c) and h was observed. Consequently, eIF3-subunits may rather be involved in benign endometrial conditions than malignancy.

In the present study, however, overexpression of eIF4g in EC was independently associated with poor prognosis. Three and 5-year survival rates for patients with tumours expressing high levels of eIF4g CSs were 71.4% and 63.8%, as compared with 81.7% and 76.2% for patients with weak-staining tumours. eIF4g is part of the mammal eIF4F complex, which induces cap-dependent translation by guiding ribosomes to the capped end of the mRNA [18]. It is built up by the ATP-dependent RNA helicase eIF4a and cap-binding protein eIF4e [18]. As a scaffold, eIF4g has three domains, the C-terminal domain interacts with the eIF4e-specific protein kinase MAP kinase-interacting serine/threonine-protein kinase 1 (Mnk-1) and has a binding site for eIF4a [34]. The cap-binding factor eIF4e is bound by the N-terminal domain, which additionally interacts with the poly(A) binding protein (PABP) [35]. The most important component of eIF4g is the central domain, with binding sites both for eIF3 and eIF4a [36]. Especially the interaction between eIF4g and eIF4a is essential for the recruitment, reconstruction and translation of mRNA [37]. eIF4e-sequestering proteins, such as 4E-BP1 binding protein, control the formation of the eIF4F complex. Usually, 4E-BP1 is kept in a hyperphosphorylated state by mTOR [38]. During hypoxia, the activity of mTOR is reduced, leading to activation of 4E-BP1. Consequently, eIF4e is sequestered by 4E-BP1, and cap-dependent mRNA translation is impaired [39]. In parallel, an alternative mechanism to the canonical translation initiation is promoted, allowing for the translation of internal ribosome entry site (IRES)-containing mRNAs [40]. Therefore, a change from the cap-dependent mRNA translation promoted by eIF4e towards the IRES-dependent mechanism sustained by 4E-BP1 and eIF4g occurs under hypoxic conditions [41]. Notably, mRNAs of important oncogenic proteins, including vascular endothelial growth factor (VEGF), B-cell lymphoma 2 (Bcl2) and Hypoxia-inducible factor 1-alpha (HIF1alpha), can be translated both via cap- and IRES-dependent mechanisms [42,43]. Consequently, overexpression of eIF4g promotes angiogenesis and tumour growth, as observed in human inflammatory breast cancer [30]. The prognostic significance of eIF4g has also been observed for nasopharyngeal carcinoma [44], with high levels being positively correlated to tumour progression. Moreover, eIF4g is frequently overexpressed in squamous cell lung carcinoma, where it is associated with amplifications in the 3q26-27 chromosomal region [45].

Interestingly, in the present study, we additionally observed a strong correlation between eIF4g expression and type of EC, with significantly higher scores found in type II EC. This may reflect the fact that eIF4g induces expression of E-cadherin, a marker typically found in type II EC [30,46]. We assume that high eIF4g levels may be necessary to sustain the characteristics of this subtype by promoting the expression of E-cadherin. However, our hypothesis warrants further in-depth research in order to correlate E-cadherin with eIF4g expression levels in EC, which is beyond the scope of the current study. Notably, the independent prognostic significance of eIF4g was lost by adding tumour type to the multivariate model, due to the strong correlation between both factors.

A similar observation regarding tumour type and expression of distinct eIFs has been made by Heikkinen et al. High eIF4e expression-levels correspond with hormone-receptor negative breast cancer subtypes, but HER2-positive subtypes [32].

## 4. Materials and Methods

### 4.1. Patient Samples

Two-hundred-seventy-nine patients diagnosed with EC between January 2000 and September 2010 at a single institution (Medical University of Gdansk) were included in this retrospective study. Patients must have been diagnosed with EC based on histopathological analysis. Patients were excluded if no follow-up was available.

A total of 1180 FFPE samples from primary tumour were analysed, with an average of four samples deriving from each patient. Fifteen endometrial mucosa samples from healthy individuals served as the control group. With a given effect size of 0.6 and anticipated standard deviation of 1.0 between NNT and EC, power-analysis with Z-statistic revealed that with a power of 0.8 a difference of 5% (alpha) between both groups can be detected, assuming that at least 28 samples are included in NNT and 119 in EC group. Due to initial built-up of the study, however, 15 patients rather than 28 were included in the NNT-group. This limitation has additionally been added as one of the limitations of the study.

The study has been approved by the Ethics Committee of the Medical University of Gdansk, with the following IRB, approval Code: NKEBN/269/2009 (09/09), approved on 14 September 2009. All patients had signed a written informed consent that tissue samples may be used for scientific purposes.

### 4.2. TMA Construction

Two experienced observers reviewed tumours and marked the relevant areas with tumours on the haematoxylin and eosin (HE)-stained sections. Out of these tumour areas, tissue arrays (1.5 mm in diameter) were punched out and embedded into a paraffin block in a predefined pattern. Subsequently, tissue sections were cut (4 µm) and fixated on specific adhesive-coated glass slides suitable for immunohistochemical staining. Both tumour grade and type were diagnosed histologically according to the current WHO classification [47], the tumour stage according to UICC. Grade 3 EC was automatically classified as type II EC.

### 4.3. Immunohistochemistry (IHC)

**Figure 4:**
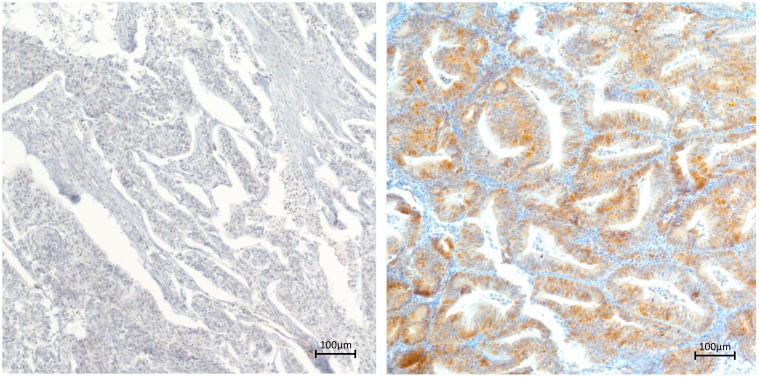
Representative immunohistochemical staining of eIF4g in endometrial carcinoma. Left panel showing low cytoplasmic eIF4g staining. Right panel showing high cytoplasmic eIF4g staining (magnification: ×10).

IHC was performed on a Ventana Immunostainer XT (Ventana Medical Systems, Tucson, AZ, USA), using an ultra-VIEW Universal DAB Detection Kit (Ventana Medical Systems, Tucson, AZ, USA) and cell conditioning solution for 30 min using heat-induced epitope retrieval (HIER). The primary antibodies were incubated for 30 min using different dilutions (Supplementary Table S2). On average, four samples of each tumour were analysed. The TMAs were scored by a research assistant and a consultant pathologist using light microscopy. For each sample, immunostaining of eIF2α, eIF3c, eIF3h, eIF4e, eIF4g, eIF5 and 4IF6 was assessed (Figure 4).

Based on visual estimation, cytoplasmic staining intensity was scored from zero to three (0 = negative, 1 = weak, 2 = moderate, 3 = strong). Cytoplasmic staining density was classified from one to four, based on the percentage of stained cells (0%–25% =1, 25–50% =2, 50–75% =3, 75–100% =4). A combined score (CS) was subsequently calculated by multiplying staining density and intensity (resulting in a maximum value of 12), with the mean value of samples per patient taken for further analyses. In the case of poor staining quality of a certain sample, it was labelled as “non-classifiable” and excluded from statistical analysis.

### 4.4. Protein Extraction and Immunoblot

Tumour samples obtained during surgery were frozen in liquid nitrogen immediately after resection and stored at −80 °C until further processing. Subsequently, samples were homogenized with MagNA Lyser homogenizer (Roche Diagnostics, Risch-Rotkreuz, Switzerland) and underwent lysis in NP-40 Lysis buffer (0.05 M Tris-HCl, 5 mM NaCl, 0.5% NP-40, 0.1 mM Pefabloc, 1 mM DTT, complete Mini, PhosSTOP). In order to determine protein concentration, the Bradford protein assay (orad Protein Assay Dye Reagent, 500-0006, BioRad Laboratories GmbH, Munich, Germany) was used. Thereafter, 30 µg of protein each were loaded onto SDS-PAGE gels (30% Acrylamid/ Bisacrylamid solution, ROTH, Karlsruhe, Germany), underwent electrophoresis in Mini-vertical electrophoresis units (Hoefer Inc, Richmond, USA) and blotted onto PVDF membranes (Immobilin-P Transfer Membrane, Millipore, Massachusetts, USA) with Semi-Dry Blotting Units (SCIE-PLAS, Cambridge, England). Membranes were blocked in TBS tween (TBST) for 1 h at room temperature with 5% non-fat milk (AppliChem, Darmstadt, Germany). Dilution of primary antibodies as visible in Supplementary Table S3 was performed in TBST and 5% BSA at 4 °C overnight. Following washing of the membranes with TBST, they were incubated with a horseradish peroxidase-conjugated secondary antibody (anti-rabbit 1:5000, GE Healthcare Life Sciences, Buckinghamshire, England). Visualization of proteins was performed by using chemiluminescence ECL kit (GE Healthcare Life Sciences). Thereafter, they were exposed on the Image Quant LAS 500 (GE Healthcare, Little Chalfont, UK) and the signal subsequently normalized with anti-β-actin antibody (mAb dilution 1:1000, Sigma-Aldrich, St Louis, MO, USA).

### 4.5. Statistical Analysis

Statistical analysis was carried out using Stata 15.1 (StataCorp, College Station, TX, USA). Demographic and tumour-related information was summarised with descriptive and explorative analysis. The mean value of all samples deriving from one patient (4 samples on average) was calculated and used for statistical analysis, resulting in 279 cases.

Median if CS were compared with Mann-Whitney-*U*- and Kruskal-Wallis-tests (tied observations). Follow-up time was measured from the date of surgery to the last appointment or date of death. Overall survival was determined as survival from resection of the tumour to the date of death. Time-to-event-analyses were calculated with Kaplan-Meier survival curves. The influence of eIF CS expression levels (subdivided into “low” (value ≤ 11 and “high” value > 11) staining groups) on overall-survival was assessed with univariate Cox-regression models. Multivariate Cox-regression models were constructed to assess the independent prognostic significance of parameters. Corresponding hazard ratios (HRs) and 95% confidence intervals (95%CI) were given. A two-sided *p*-value of less than 0.05 was considered statistically significant.

### 4.6. Ethics Statement

The study was accepted by the Independent Ethics Committee of the Medical University of Gdańsk (NKEBN/269/2009). Procedures involving human subjects were in accordance with the Helsinki Declaration of 1975, as revised in 1983.

## 5. Conclusions

In summary, we identified eIF4g as an independent prognostic factor in endometrial carcinoma, irrespective of tumour stage and patient age. The strong correlation between eIF4g and endometrial cancer subtype, as observed in the present study, warrants further investigations, though. Considering the limitations of traditional subclassification of EC into type I and type II [8], eIF4g could well serve as an alternative prognostic marker, independently indicating a poor patient prognosis.

## Figures and Tables

**Table 1:** Characterization of demographic and tumour-related parameters.

		Count	%	Missing
Age	<60 years	117	41.9	0
	>60 years	162	58.1	
EC Type	Type I	216	90.0	39
	Type II	24	10.0	
Grading	G1	134	48.0	0
	G2	107	38.4	
	G3	38	13.6	
Staging	I	184	67.4	5
	II	42	15.2	
	III	34	12.3	
	IV	14	5.1	
NNT		15	100.0	0

**Table 2:** Differences in eIF combined scores (CS) with given medians and interquartile ranges (IQR).

		eIF2a CS		eIF3c CS		eIF3h CS		eIF4e CS	
		Median (IQR)	*p*-Value	Median (IQR)	*p*-Value	Median (IQR)	*p*-Value	Median (IQR)	*p*-Value
Tissue	NNT	12 (12–12)	0.317	12 (12–12)	0.129	12 (12–12)	0.304	8 (8–8.9)	0.463
	EC	12 (12–12)		12 (9.3–12)		12 (12–12)		8 (5.3–9.1)	
Age	<60 years	12 (12–12)	0.051	12 (9.3–12)	0.147	12 (12–12)	0.948	8.4 (5–11.1)	0.909
	>60 years	12 (12–12)		12 (10–12)		12 (12–12)		8 (6–11)	
EC Type	Type I	12 (12–12)	0.771	12 (9.3–12)	0.906	12 (12–12)	0.770	8 (5.7–11)	0.566
	Type II	12 (12–12)		12 (9.3–12)		12 (12–12)		8 (6–10)	
Grading	NNT	12 (12–12)	0.722	12 (12–12)	0.623	12 (12–12)	0.413	8 (8–8)	0.835
	G1	12 (12–12)		12 (9.3–12)		12 (12–12)		8 (5–11)	
	G2	12 (12–12)		12 (10–12)		12 (12–12)		9 (6–11)	
	G3	12 (12–12)		12 (8–12)		12 (12–12)		8 (5–12)	
Staging	NNT	12 (12–12)	0.442	12 (12–12)	0.578	12 (12–12)	0.609	8 (8–12)	0.214
	I	12 (12–12)		12 (12–12)		12 (12–12)		8 (5–11)	
	II	12 (12–12)		12 (12–12)		12 (12–12)		9 (6–11)	
	III	12 (12–12)		12 (12–12)		12 (12–12)		10 (7–12)	
	IV	12 (12–12)		12 (12–12)		12 (12–12)		9 (6–12)	

Significance tested with Mann-Whitney-*U*-test (two groups) or Kruskal-Wallis-test (>two groups).

**Table 3:** Differences in eIF combined scores (CS) with given medians and interquartile ranges (IQR), significance tested with Kruskal-Wallis-tests (tied observations).

		eIF4g CS		eIF5 CS		eIF6 CS	
		Median (IQR)	*p*-Value	Median (IQR)	*p*-Value	Median (IQR)	*p*-Value
Tissue	NNT	8 (8–12)	0.854	4 (4–4)	**<0.001**	12 (12–12)	**<0.001**
	EC	10 (8–12)		8 (5–10.7)		10 (8–12)	
Age	<60 years	10 (7–12)	0.800	8 (4.7–10.7)	0.826	11 (8–12)	0.071
	>60 years	10 (7–12)		8 (5–10.7)		10 (8–12)	
EC Type	Type I	10 (6.7–12)	**0.034**	8 (5–10)	0.681	10 (8–12)	0.763
	Type II	11 (9.8–12)		7 (5–12)		10 (9–12)	
Grading	NNT	8 (8–8)	**0.014**	4 (4–4)	**<0.001**	12 (12–12)	**0.032**
	G1	9 (6–12)		7 (4–10)		10.7 (8–12)	
	G2	10.8 (8–12)		8 (5–11)		10 (8–12)	
	G3	11 (7–12)		9 (7–12)		11.7 (8.1–12)	
Staging	NNT	8 (8–12)	0.315	4 (4–4)	**0.004**	12 (12–12)	**0.020**
	I	10 (7–12)		8 (4–11)		10 (8–12)	
	II	9 (6.7–12)		8 (5.3–10)		10 (8–12)	
	III	11 (9–12)		7 (5–10)		11 (8–12)	
	IV	12 (5.5–12)		8 (6–12)		11 (7–12)	

Significance tested with Mann-Whitney-*U*-test (two groups) or Kruskal-Wallis-test (>two groups); bold = significant results.

**Table 4:** Univariate Cox-regression analysis evaluating the prognostic influence of eIFs on overall survival.

		HR	95% Confidence Interval		*p*-Value
			Lower	Upper	
eIF2α	Low	1			0.906
	High	1.039	0.552	1.953	
eIF3c	Low	1			0.484
	High	1.168	0.756	1.805	
eIF3h	Low	1			0.681
	High	1.208	0.490	2.977	
eIF4e	Low	1			0.842
	High	0.952	0.584	1.551	
eIF4g	Low	1			**0.013**
	High	1.687	1.116	2.550	
eIF5	Low	1			0.708
	High	1.098	0.674	1.790	
eIF6	Low	1			0.934
	High	1.018	0.671	1.542	

Legend: bold = significant result.

**Table 5:** Multivariate Cox-regression analysis showing the independent prognostic impact of eIF4g expression on overall survival.

		HR	95% Confidence Interval		*p*-Value
			Lower	Upper	
eIF4g	Low	1			**0.034**
	High	1.604	1.037	2.483	
Age	Continuous	1.086	1.060	1.112	**<0.001**
Stage	I	1			
	II	1.524	0.833	2.789	0.172
	III	3.739	2.110	6.623	**<0.001**
	IV	8.969	4.608	17.456	**<0.001**

Legend: bold = significant result.

## Supplementary Materials

Supplementary materials can be found at https://www.mdpi.com/1422-0067/20/24/6169/s1.

## Abbreviations

EC	Endometrial cancer
CS	Combined score
eIF	Eukaryotic translation initiation factor
NNT	Non-neoplastic tissue
PIK3CA	Phosphatidylinositol-4,5-bisphosphate 3-kinase catalytic subunit alpha
PTEN	Phosphatase and Tensin homolog
mTOR	Mechanistic Target of Rapamycin
RNA	Ribonucleic acid
NF-kB	Nuclear factor “kappa-light-chain-enhancer” of activated B-cells
SD	Standard deviation
HR	Hazard ratio
95%CI	95% Confidence interval
IHC	Immunohistochemistry
mRNA	Messenger RNA
ATP	Adenosine triphosphate
MAP	Mitogen-activated protein
Mkn-1	MAP kinase-interacting serine/threonine-protein kinase 1
PABP	Poly(A) binding protein
IRES	Internal ribosome entry site
VEGF	Vascular endothelial growth factor
Bcl2	B-cell lymphoma 2
HIF1alpha	Hypoxia-inducible factor 1-alpha
HER2	Human epidermal growth factor receptor 2
FFPE	Fresh-frozen paraffin-embedded
TMA	Tissue micro-array
WHO	World Health Organization
UICC	Union International Contre le Cancer
