# Supplementary material for: The Prognostic Significance of Eukaryotic Translation Initiation Factors (eIFs) in Endometrial Cancer
Source: Int J Mol Sci. 2019 Dec 6;20(24):6169. doi: 10.3390/ijms20246169 (PMC6941158; doi:10.3390/ijms20246169)
Supplement: Supplementary file 1 [file ijms-20-06169-s001.zip › ijms-646167-supplementary/Supplementary Figure S1.docx]

**
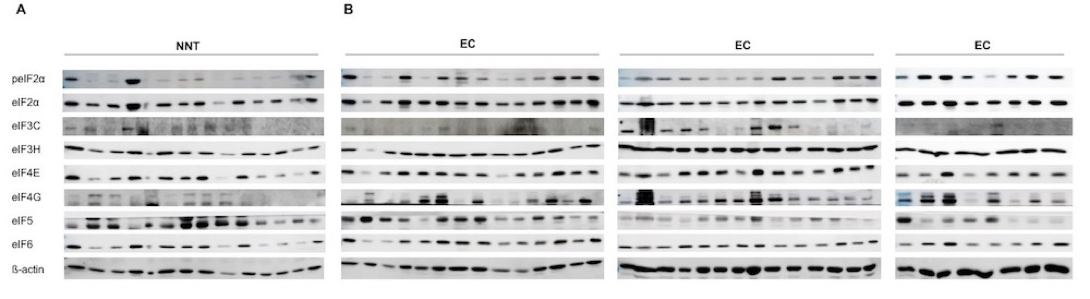
**

**Supplementary Figure S1.** eIF protein expression in in endometrial carcinoma. [A] Immunoblot analyses from non-neoplastic tissues (NNT). Equal amounts of protein were resolved on SDS PAGE and immunoblotted with eIF subunits p2α, 2α, 3C, 3H, 4E, 4G, 5, 6, and β-actin (loading control) antibodies. [B] Immunoblot analyses from endometrial carcinoma. Equal amounts of protein were resolved on SDS PAGE and immunoblotted with eIF subunits p2α, 2α, 3C, 3H, 4E, 4G, 5, 6, and β-actin (loading control) antibodies.
