# Supplementary material for: The Prognostic Significance of Eukaryotic Translation Initiation Factors (eIFs) in Endometrial Cancer
Source: Int J Mol Sci. 2019 Dec 6;20(24):6169. doi: 10.3390/ijms20246169 (PMC6941158; doi:10.3390/ijms20246169)
Supplement: Supplementary file 1 [file ijms-20-06169-s001.zip › ijms-646167-supplementary/Supplementary Table S1.docx]

| Supplementary Table S1. Correlation between the different eIFs. | | | | | | | | | | |
| --- | --- | --- | --- | --- | --- | --- | --- | --- | --- | --- |
|  |  | **eIF2a** | **eIF3c** | | **eIF3h** | **eIF4e** | **eIF4g** | **eIF5** | | **eIF6** |
| **eIF2a** | *Correlation coefficient* | 1 | ,359** | | ,475** | ,292** | ,240** | ,290** | | ,238** |
|  | *p-value (2-sided)* |  | **<0.005** | | **<0.005** | **<0.005** | **<0.005** | **<0.005** | | **<0.005** |
|  | *n=* | 284 | 284 | | 284 | 284 | 284 | 284 | | 284 |
| **eIF3c** | *Correlation coefficient* | ,359** | 1 | | ,313** | ,138* | ,209** | ,149* | | ,259** |
|  | *p-value (2-sided)* | **<0.005** |  | | **<0.005** | **0,018** | **<0.005** | **0,011** | | **<0.005** |
|  | *n=* | 284 | 296 | | 296 | 295 | 296 | 295 | | 295 |
| **eIF3h** | *Correlation coefficient* | ,475** | ,313** | | 1 | ,204** | ,194** | 0,087 | | ,179** |
|  | *p-value (2-sided)* | **<0.005** | **<0.005** | |  | **<0.005** | **<0.005** | 0.138 | | **<0.005** |
|  | *n=* | 284 | 296 | | 296 | 295 | 296 | 295 | | 295 |
| **eIF4e** | *Correlation coefficient* | ,292** | ,138* | | ,204** | 1 | ,311** | ,155** | | ,210** |
|  | *p-value (2-sided)* | **<0.005** | **0.018** | | **<0.005** |  | **<0.005** | **0.008** | | **<0.005** |
|  | *n=* | 284 | 295 | | 295 | 295 | 295 | 295 | | 295 |
| **eIF4g** | *Correlation coefficient* | ,240** | ,209** | | ,194** | ,311** | 1 | ,150** | | ,268** |
|  | *p-value (2-sided)* | **<0.005** | **<0.005** | | **<0.005** | **<0.005** |  | **0.01** | | **<0.005** |
|  | *n=* | 284 | 296 | | 296 | 295 | 296 | 295 | | 295 |
| **eIF5** | *Correlation coefficient* | ,290** | ,149* | | 0,087 | ,155** | ,150** | 1 | | ,207** |
|  | *p-value (2-sided)* | **<0.005** | **0.011** | | 0.138 | **0.008** | **0.01** |  | | **<0.005** |
|  | *n=* | 284 | 295 | | 295 | 295 | 295 | 295 | | 295 |
| **eIF6** | *Correlation coefficient* | ,238** | ,259** | | ,179** | ,210** | ,268** | ,207** | | 1 |
|  | *p-value (2-sided)* | **<0.005** | | **<0.005** | **<0.005** | **<0.005** | **<0.005** | | **<0.005** |  |
|  | *n=* | 284 | | 295 | 295 | 295 | 295 | | 295 | 295 |
| *This correlation is significant at 0.01; **This correlation is significant at 0.05 | | | | | | | | | | |
