# Supplementary material for: The Prognostic Significance of Eukaryotic Translation Initiation Factors (eIFs) in Endometrial Cancer
Source: Int J Mol Sci. 2019 Dec 6;20(24):6169. doi: 10.3390/ijms20246169 (PMC6941158; doi:10.3390/ijms20246169)
Supplement: Supplementary file 1 [file ijms-20-06169-s001.zip › ijms-646167-supplementary/Supplementary Table S2.docx]

| Supplementary Table S2. Dilutions of antibodies used for eIF2a, eIF3c, eIF3h, eIF4e, eIF4g, eIF5 and eIF6 immunostaining. | | | |
| --- | --- | --- | --- |
| **Primary Antibody** | **Company** | **Dilution** | **Second Antibody** |
| eIF4G | Cell Signaling (#2498) | 1:25 | Rabbit |
| eIF4E | Cell Signaling (#9742) | 1:100 | Rabbit |
| eIF2α (D7D3) XP | Cell Signaling (#5324) | 1:2000 | Rabbit |
| eIF3c | Santa Cruz (sc-74507) | 1:250 | Mouse |
| eIF6 | Gene Tex (GTX63642) | 1:75 | Rabbit |
| eIF5 | Santa Cruz (sc-133541) | 1:50 | Rabbit |
| eIF3H (D9C1) XP | Cell Signaling (#3413) | 1:750 | Rabbit |
